# Modelling the Thin-Layer Drying Kinetics of Marinated Beef during Infrared-Assisted Hot Air Processing of Biltong

**DOI:** 10.1155/2021/8819780

**Published:** 2021-02-09

**Authors:** Francis C. Muga, Moses O. Marenya, Tilahun S. Workneh

**Affiliations:** ^1^Department of Bioresources Engineering, School of Engineering, University of KwaZulu-Natal, Private Bag X01, Pietermaritzburg, South Africa; ^2^Department of Agricultural and Rural Engineering, School of Agriculture, University of Venda, Private Bag X5050, Thohoyandou, South Africa

## Abstract

Biltong is a dried meat product that is widely consumed in South Africa. The marinated meat is traditionally dried under ambient winter conditions while commercial biltong producers use hot air driers. Hot air drying is time-consuming and energy-intensive. A combined infrared and hot air drying (IRHAD) is an alternative method of drying meat during biltong processing. The aim of this study was to establish the effect of the infrared (IR) power, the temperature, and velocity of the drying air on the drying kinetics of marinated beef and subsequently select the best thin-layer drying model for IRHAD during biltong processing. Marinated beef samples were dried at IR power levels of 500, 750, and 1000 W; drying air temperatures of 30, 35, and 40°C; and air velocity of 1.5 and 2.5 m∙s^−1^. Results indicate that increasing the IR power and the drying air temperature increased the IR emitter temperature and the core temperature of the marinated beef sample. Consequently, increasing the drying rate thus reduced drying time. The air velocity had an inverse relationship with the IR emitter temperature, the core temperature of the marinated beef sample, and the drying rate. The drying process was characterised by a rising rate period in the first half an hour, followed by a falling rate period which implies that moisture transport occurred partly by surface evaporation and predominantly by diffusion. The effective moisture diffusivity ranged from 4.560 × 10^−10^ to 13.7 × 10^−10^ m^2^∙s^−1^, while, the activation energy ranged between 40.97 and 59.16 kJ∙mol^−1^. The IRHAD of marinated beef during its processing to biltong was best described by the two-term model since it had the highest *R*^2^ (0.9982-0.9993) and the lowest RMSE (0.0062-0.0099). The power level of the IR emitter of 1000 W combined with a drying air temperature and velocity of 40°C and 1.5 m∙s^−1^, respectively, showed the highest improvement in the drying kinetics and the lowest drying time of 5.61 ± 0.35 hours; hence, it is recommended as a possible drying alternative for the processing of biltong.

## 1. Introduction

Biltong is a dried meat product that is predominantly consumed in South Africa. Biltong is made by drying thin slices of meat that is marinated with a mixture of salt, vinegar and dry spices (coriander, black pepper, and brown sugar) [1]. The marinated slices of meat are traditionally dried under ambient conditions during the winter season (temperature < 20°C), whereas commercial biltong producers use temperature-controlled hot air dryers with the drying air temperature ranging between 20 and 40°C ([2, 3]; Burfoot *et al.*, 2010; [4]).

The market value of biltong in South Africa was estimated at US$ 170 million in 2015 [5]. The market for biltong has expanded both locally and internationally prompting its increased production [6, 7]. The rising popularity of biltong has spurred the development of a variety of hot air biltong dryers. The hot air biltong dryers range from simple domestic types to high capacity commercial dryers.

The conventional hot air drying (HAD) method used in the commercial production of biltong is an energy-intensive drying method [8]. The low thermal conductivity of agricultural products combined with the case hardening of these products during HAD decelerates the moisture migration which results in longer drying time and increased energy consumption [9, 10]. HAD degrades the quality of agricultural products through the loss of colour, loss of heat-sensitive nutrients, deformation, and internal structure damage [11–13]. Moreover, the temperature range of 20–40°C used in conventional HAD of meat during biltong production is not sufficient to achieve the recommended microbial reduction in the resultant biltong [3]. These concerns underscore the need for alternative drying methods for biltong production.

Alternative heating technologies like microwave, inductive heating, radiofrequency, and infrared (IR) provide volumetric heating that positively impacts on the energetic, exergetic, and heating efficiency [14]. According to Li et al. [15], IR radiation improves the dehydration efficiency of beef jerky by promoting protein denaturation which transforms immobilised water to free water, consequently reducing the activation energy. Cherono et al. [16] noted that IR drying of beef reduced the microbial count on the resulting biltong. However, moisture condensation observed on the surface of biltong during IR drying highlights the inability of natural convection to cope with the improved drying rates [17]. A combined infrared and hot air drying (IRHAD) could accelerate the removal of moisture from the meat surface to sustain the high drying rates. Drying agricultural products using IRHAD requires less energy and produces dried products of higher quality compared to using IR drying or HAD independently [18, 19].

The application of IRHAD as a possible alternative to HAD in the making of biltong requires a quantitative understanding of the heat and mass transfer in meat subjected to IRHAD. According to Trujillo et al. [20], Fickian diffusion is the predominant mode of moisture transport in meat during drying. The Page model and thin layer models derived from Fick's Second Law of diffusion suitably describe the drying behaviour of agricultural products. Abe and Afzal [21] and Das et al. [22] identified the Page model as the best model for describing the thin-layer IR drying of rough rice. According to Toğrul [23], Puente-Díaz et al. [24], Sui et al. [25], and Sadin et al. [26], the Midilli model is the most suitable model for predicting the drying kinetics of apple, murta berries, wine grape pomace, and tomato slices, respectively, when subjected to IRHAD. The Logarithmic model was identified by Doymaz [27] as the best model for the IR drying of sweet potatoes. The study by Cherono [17] identified the approximation of diffusion model as the best model for the IR drying of marinated beef. Nonetheless, the two-term model and the Midilli model also showed good accuracy in predicting the IR drying of marinated beef.

A complete characterisation of the drying kinetics is critical in modelling the drying process [28]. The aforementioned studies highlight the research done in modelling the drying of a number of agricultural products subjected to IR drying and IRHAD. However, there is no literature on the drying characteristics of marinated beef subjected to IRHAD. A good understanding of the drying kinetics of marinated beef subjected to IRHAD would guide the application of IRHAD as a possible alternative to HAD in the processing of biltong. Therefore, the aim of this study was to establish the effect of the IR power, the temperature, and velocity of the drying air on the drying kinetics of marinated beef and subsequently select the best thin-layer drying model.

## 2. Materials and Methods

### 2.1. Sample Preparation

The samples were prepared as outlined by Muga et al. [1]. Beef portions procured from a local butchery (Pick n Pay, Pietermaritzburg, South Africa) were sliced along the muscle fibres to dimensions of 150 × 50 × 15 mm [2]. The moisture content of each sample was determined by drying a 10 g portion of the sample at 105°C for 24 hours in a hot air oven (AX 60, Carbolite Gero Ltd., Hope Valley, UK) [29]. The experimental samples were put in sealed polythene bags and stored in a refrigerator (Defy C250, Defy Appliances (Pty) Ltd., Durban, South Africa) set at 4°C, awaiting marination.

The biltong marinade was made by mixing sodium chloride and vinegar in a ratio of 1 : 2, respectively, based on their mass. The experimental beef samples were marinated by adding 0.075 kg of marinade for every kg of beef [30, 31]. The marinade was uniformly spread over the beef samples to ensure that all sides of the beef samples were coated with the marinade. The marinated samples were refrigerated at 4°C, for 24 hours. The samples were turned every six hours during the refrigerated storage period to ensure uniform distribution of marinade [32].

### 2.2. The Drying Unit

**Figure 1:**
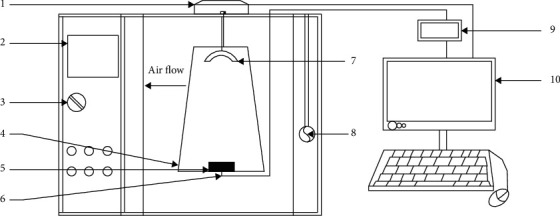
Schematic of the drying cabinet. 1: Weighing balance; 2: digital control panel; 3: power knob; 4: drying platform; 5: beef sample; 6: K-type thermocouple; 7: infrared emitter; 8: anemometer; 9: temperature datalogger; 10: computer monitor.

The infrared-assisted hot air dryer was made by retrofitting an existing hot air cabinet dryer with an IR emitter. A schematic of the IRHAD experimental set-up is shown in Figure 1. The drying chamber is made of stainless steel and has a 100 mm thick polyurethane insulation. A drying platform (200 × 100 mm) made of stainless steel mesh is suspended on a weighing balance (Shimadzu UW6200H, Shimadzu Corporation, Kyoto, Japan) that is placed on top of the drying cabinet. The weighing balance is connected to a computer via an RS-232C connector to log the mass of the sample being dried. A complete description of the hot air cabinet drier is provided in [1].

A ceramic IR emitter (T-FSR, Elstein-Werk M. Steinmetz GmbH & Co. KG, Northeim, Germany) is attached to the roof of the drying chamber, directly above the drying platform (Figure 1). The distance between the IR emitter and the drying platform is 280 mm. The IR emitter has a power rating of 1000 W and is of dimensions 250 by 62.5 mm. The wavelength of the IR radiation from the IR emitter ranges from 2 to 10 *μ*m [33]. The power to the IR emitter was varied between 500 and 1000 W by regulating the current using an AC light dimmer circuit. The IR emitter has an inbuilt K-type thermocouple that was connected to a data logger (OM-DAQ-USB-2401, Omega, UK) to record its temperature.

The temperature and velocity of the drying air are monitored using a supervisory control and data acquisition (SCADA) system that is installed in the dryer. The core temperature of the sample placed on the drying platform is measured using a K-type thermocouple (Temperature Controls (Pty), Pinetown, South Africa). The K-type thermocouple is connected to a data logger (OM-DAQ-USB-2401, Omega, UK) to record the core temperature data.

### 2.3. Drying Experiments

A marinated beef sample was retrieved from refrigerated storage and allowed to equilibrate to room temperature for 60 minutes. Synchronously, the dryer was preheated for 60 minutes before the commencement of the drying experiments. A five-gram portion of meat was cut from the beef sample for the determination of the moisture content prior to drying [29]. Thereafter, a K-type thermocouple was inserted into the centre of the beef sample, and the sample was placed on the drying platform as shown in Figure 1. The IR emitter was instantaneously switched on, and the beef sample allowed to dry until 50% of its mass was lost. The core temperature and mass of the beef sample, as well as the temperature of the IR emitter, were recorded continuously throughout the drying experiment.

The drying experiment employed a three-factor, full-factorial experimental design. The first two factors had three levels, and the third factor had two levels. The factors studied were the IR power (500, 750, and 1000 W), the temperature of the drying air (30, 35, and 40°C), and the velocity of the drying air (1.5 and 2.5 m.s^−1^).

The data obtained from the experiments was subjected to the analysis of variance (ANOVA) at 5% level of significance. Where a significant ANOVA result was found, the mean comparison was done using Fisher's Unprotected LSD method. All data analysis was done using GenStat® 18^th^ Edition (VSN International Ltd., Hemel Hempstead, United Kingdom).

### 2.4. Evaluation of the Drying Characteristics

The moisture loss data obtained from the drying experiments was used to determine the instantaneous moisture content of the beef sample during drying. Thereafter, the moisture ratio (MR) was calculated using equation (1), and the drying rate (*D*_*R*_) was obtained from the derivative of the MR with respect to drying time (equation (2)).

(1) $$\begin{array}{c} \text{MR}=\frac{X_{t}}{X_{0}}, \end{array}$$

(2) $$\begin{array}{c} D_{R}=\frac{d\text{MR}}{dt}, \end{array}$$

where:

*t* = drying time (s)

*X*_*t*_ = instantaneous moisture content (kg of water/kg of dry matter)

*X*_0_ = the initial moisture content (kg of water/kg of dry matter)

The effective moisture diffusivity (*D*_eff_) was obtained from the linear form of the solution to Fick's Second Law of diffusion as shown in equation (3) [34].

(3) $$\begin{array}{c} \ln\text{ MR}=\ln\frac{8}{\pi^{2}}-{\left(\frac{\pi }{2L}\right)}^{2}D_{\text{eff}}t, \end{array}$$

where:

*L* = the half-thickness of the beef sample (m)

The activation energy (*E*_*a*_) was determined from the Arrhenius-type relationship between the drying temperature and the effective moisture diffusivity (equation (4)) [35].

(4) $$\begin{array}{c} D_{\text{eff}}=D_{o}\exp\left(-\frac{E_{a}}{RT}\right), \end{array}$$

where:

*D*_0_ = the preexponential factor of Arrhenius equation equivalent to diffusivity at the maximum temperature (m^2∙^s^−1^)

*E*_*a*_ = the activation energy (kJ^∙^mol^−1^)

*R* = the universal gas constant (kJ^∙^mol^-1∙^K^−1^)

*T* = the average beef sample temperature in (°K)

The activation energy (*E*_*a*_) was obtained from the linear form of equation (4) (equation (5)) by determining the slope of the graph of ln(*D*_eff_) against (−1/RT).

(5) $$\begin{array}{c} \ln D_{\text{eff}}=\left(-\frac{1}{\text{RT}}\right)E_{a}+\ln D_{\text{o}}. \end{array}$$

### 2.5. Selection of the Best Thin-Layer Drying Model

The thin-layer drying models considered in this study are outlined in Table 1. The models were selected based on previous research as highlighted in the introduction.

The experimental data was fitted to the selected thin layer dying models using the nonlinear least square analysis in MATLAB (MATLAB R18.2b, Mathworks, Inc., Natick, MA, USA). The best model was chosen based on a combination of the highest *R*^2^ and the lowest RMSE.

## 3. Results and Discussion

### 3.1. IR Emitter Temperature

The temperature of the IR emitter recorded for the tested drying conditions is presented in Table 2. The temperature of the IR emitter was significantly (*p* ≤ 0.05) affected by the power level of the IR emitter. The highest and lowest IR emitter temperatures of 566.51 ± 3.6°C and 219.03 ± 2.62°C were observed at an IR power level of 1000 W and 500 W, respectively. Increasing the IR emitter power consumption increased the temperature of the IR emitter. The increase in the emitter temperatures is caused by the increase in ohmic losses as the power level of the IR emitter increases [9]. These results are consistent with previous findings by Ali et al. [39] and Ott et al. [40].

A change in the velocity of the drying air resulted in significant (*p* ≤ 0.05) variation in the temperature of the IR emitter. Higher IR emitter temperatures were observed at a low drying air velocity of 1.5 m∙s^−1^, while the drying air velocity of 2.5 m∙s^−1^ resulted in low IR emitter temperatures. The reduction in the temperature of the IR emitter at higher drying air velocity is attributed to the cooling effect induced by the drying air on the IR emitter due to convective heat losses. According to Das and Das [41], the radiation efficiency of mid to far IR emitters ranges between 40 and 60% with some heat lost via convection. Increasing the drying air velocity increases the mass flow rate of air which increases the convective heat losses, thus reducing the IR emitter temperature.

The temperature of the drying air also had a significant (*p* ≤ 0.05) effect on the temperature of the IR emitter. The temperature of the IR emitter increased with an increase in the drying air temperature. An increase in the drying air temperature reduced the temperature gradient between the IR emitter and the drying air, thus reducing the convective heat losses and vice versa. Consequently, the IR emitter temperatures increased with an increase in drying air temperature.

The power level of the IR emitter had a synergistic interaction with the temperature and velocity of the drying air that significantly (*p* ≤ 0.05) affected the temperature of the IR emitter. However, the two-way interaction between the drying air temperature and velocity, and the three-way interaction of all the experimental factors, had no significant effect on the IR emitter temperature.

### 3.2. Core Temperature of the Beef Sample during Drying

The average core temperature of the beef sample over the entire drying period ranged from 30.62 ± 0.08 to 51.16 ± 0.36°C (Table 3). The core temperature of the beef sample was significantly (*p* ≤ 0.05) affected by the power level of the IR emitter. Higher core temperatures of the sample were observed at higher power levels of IR emitter. The increase in the core temperature of the beef sample with an increasing power level of the IR emitter is caused by the high temperatures of the IR emitter achieved at high power levels of the IR emitter.

The temperature of the IR emitter determines the quality and intensity of IR radiation [42]. According to Planck's law, higher temperatures of the IR emitter shift the peak IR energy wavelengths towards the shorter wavelength [43]. The short IR wavelengths are associated with higher IR energy flux and vice versa [44]. The peak IR wavelengths calculated using the Wien's displacement law and the average temperatures of the IR emitter were 5.39 ± 0.17, 7.51 ± 0.48, and 12.73 ± 0.43 *μ*m, at 1000, 750, and 500 W, respectively. The shorter wavelength of 5.39 ± 0.17 *μ*m observed at and an IR power level of 1000 W indicates an increase in the IR energy flux into the beef sample. The increased IR energy flux into the beef sample increases the core temperature of the beef sample at the IR power level of 1000 W compared to the IR power level of 750 and 500 W. These results concur with previous research that reported an increase in the core temperature of the sample with an increase in IR power [17, 45].

The velocity of the drying air caused significant (*p* ≤ 0.05) variation in the average core temperature of the beef sample. A higher core temperature of the beef samples was observed at a drying air velocity of 1.5 m∙s^−1^, whereas lower core temperatures were observed at a drying air velocity of 2.5 m∙s^−1^. An increase in the air velocity is associated with low IR emitter temperature which reduces the radiant energy flux into the sample, resulting in low core temperature of the samples. The inverse relationship between the drying air velocity and the core temperature of the beef sample can also be attributed to the evaporative cooling due to the increased mass flow rate of the drying air as observed by Kocabiyik and Tezer [46] during the IR drying of carrots.

**Figure 2:**
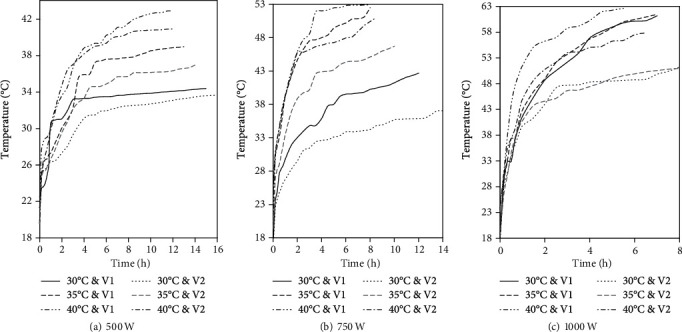
Variation in core temperature of the beef sample during drying at selected IR emitter power temperature and air velocity of the drying air (V1 = 1.5 m∙s^−1^ and V2 = 2.5 m∙s^−1^).

The core temperature of the beef sample also varied significantly (*p* ≤ 0.05) with the temperature of the drying air. Increasing the temperature of the drying air increased the core temperature of the sample. The variation in core temperature of the sample became pronounced over time as shown by the divergence of the sample temperature curves in the later stages of drying (Figure 2). This variation can be attributed to the synergistic transfer of energy from both the IR and the hot drying air to the beef sample from the onset of drying until the sample temperature reaches the drying air temperature [19]. Thereafter, there is simultaneous energy transfer into and out of the beef sample. The energy influx is primarily due to the radiant flux from the IR emitter while the energy outflux is driven by the temperature gradient between the sample and the drying air. The temperature gradient between the beef sample and the drying air increases with a decrease in the drying air temperature, thus promoting a high energy outflux which results in lower core temperature of the beef sample.

### 3.3. Drying Time

The drying time is the duration spent in drying the beef sample until 50% of the original mass was lost. The drying time ranged from 5.61 ± 0.35 to 16.22 ± 0.25 hours (Table 4). This is significantly lower than the 24–230 hours required to dry biltong using the conventional hot air drying [3, 4, 17, 47]. The drying time achieved in this study is also lower than the range of drying time of 10.25–36 hours, achieved by Cherono [17] that solely used IR drying to make biltong. A similar reduction in drying times has been reported by Sharma et al. [48] and Li et al. [15] on IRHAD of onions and beef jerky, respectively. According to Hebbar et al. [19], the synergistic interaction between IR and hot air promotes rapid heating of the product being dried which accelerates the rate of mass transfer, thus improving the dehydration efficiency and subsequently lowering the drying time.

**Figure 3:**
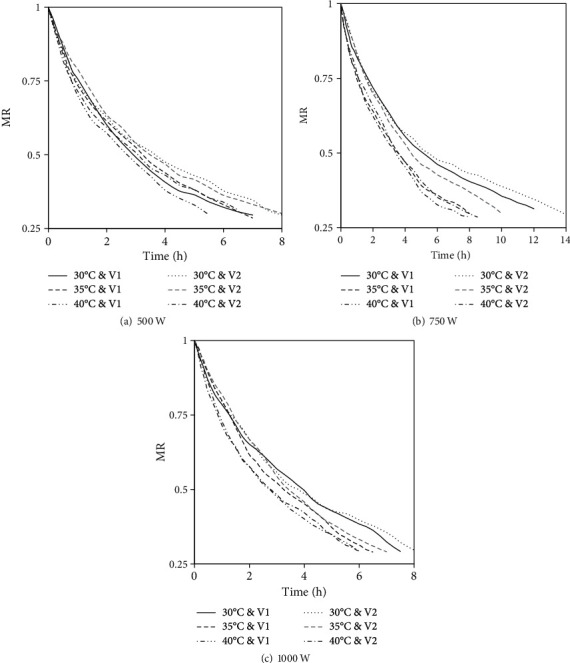
Variation in the MR of marinated beef during IRHAD.

The variation in MR over the drying period is presented in Figure 3. The IR power significantly (*p* ≤ 0.05) affected the drying time. Shorter drying times were observed at 1000 W while the longest drying time was observed at an IR power level of 500 W. Similarly, the drying time decreased significantly (*p* ≤ 0.05) with an increase in temperature of the drying air. These observations are in agreement with the findings of Sharma et al. [48], Kumar et al. [49], and Nasiroglu and Kocabiyik [50]. An increase in both the IR power and the drying air temperature increases the core temperature of the beef sample. The increase in the core temperature of the beef sample indicates the increased heat influx which accelerates the vapourisation of water in the beef sample, thus increasing the vapour pressure and expediting the moisture transport out of the sample [51]. According to Jaturonglumlert and Kiatsiriroat [52], increasing the power of the IR emitter and the temperature of the drying air increases the ratio of heat and mass transfer coefficient, thus leading to the shortening of the drying time.

Contrary to the effect of the power level of the IR emitter and the temperature of the drying air, increasing the velocity of the drying air increased the drying time. Similar findings were reported by Afzal et al. [18] and Kocabiyik and Tezer [46]. This observation can be attributed to the negative effect of the drying air velocity on the core temperature of the beef sample. Increasing the drying air velocity decreases the ratio of heat and mass transfer coefficient, thus lengthening the drying time [52].

### 3.4. Drying Rate

**Figure 4:**
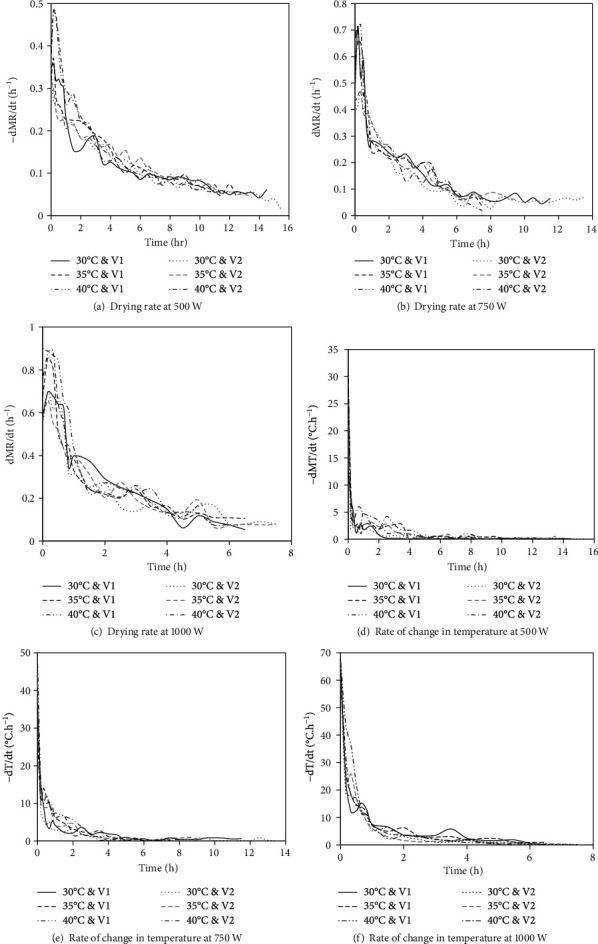
Variation in the drying rate and the rate of change in the core temperature of the beef sample during drying.

The drying rate refers to the rate of moisture removal from the beef sample. The variation in the drying rate during the drying process is shown in Figure 4. The drying rate curves at the IR power level of 500 and 750 W indicated a short rising rate drying period followed by the first and second falling rate drying periods which is typical of drying rate curves obtained from IRHAD [53]. The drying rate curve obtained at the IR power level of 1000 W showed a short constant rate drying period immediately after the rising rate period and just before the first falling rate period. This observation is consistent with the results obtained by Mongpraneet et al. [54] when drying welsh onions under IR radiation.

IR radiation has an efficient heat transfer mechanism that promotes a rapid increase in the core temperature of the beef sample at the onset of drying, thus increasing the drying rate [43]. The rising rate drying period occurred within the first 30 minutes of drying and coincided with an increase in the rate of change in the core temperature of the beef sample (Figure 4). The moisture transport during this period can be attributed to surface evaporation [53].

The rising rate drying period is superseded by the first falling rate drying period. The first falling rate drying period coincides with a decrease in the rate of change in the core temperature of the beef sample (Figure 4). This drying period occurred between 30 minutes and 6 hours of drying at IR power levels of 500 and 750 W, while the same occurred at between 30 minutes and 2 hours of drying at IR power level of 1000 W. According to Trujillo et al. [20], diffusion is the predominant mode of moisture transport during the first falling rate drying period. The decrease in drying rate can be attributed to the reduced heat flux into the sample which lowers the vapour pressure gradient and subsequently reduces the moisture transport out of the sample.

The second falling rate period occurred after the first 6 hours of drying at IR power levels of 500 and 750 W, while the same occurred after the first 2 hours of drying at an IR power level of 1000 W. The second falling rate period is characterised by a low and fluctuating drying rate. According to Kocabiyik [53], most of the free water is lost during the first falling rate period leaving the bound water to play an active role in the second falling rate period. Bound water requires more energy to extract from the food matrix. However, there is minimal heat flux into the beef sample during this drying period as indicated by the relatively constant product temperature. The low drying rate may also be attributed to the additional internal resistance to moisture movement as the sample moisture content decreases [55].

The drying rate was significantly (*p* ≤ 0.05) affected by the power level of the IR emitter, the temperature, and the velocity of the drying air. This is attributed to the effect that these factors had on the core temperature of the beef sample, consequently affecting the amount of heat flux into the sample. Feyissa et al. [56] reported that heat transfer is the key driver of mass transfer during the roasting of chicken meat. The coupled relationship between the core temperature of the beef sample and the moisture transport is illustrated by the synchrony between the drying rate curve and the curve depicting the rate of increase in sample temperature (Figure 4).

### 3.5. Effective Moisture Diffusivity

The effective moisture diffusivity (*D*_eff_) of marinated beef subjected to infrared assisted hot air drying ranged between 4.560 × 10^−10^ m^2^∙s^−1^ and 13.7 × 10^−10^ m^2^∙s^−1^ (Table 5). These values are within the range of values obtained by Li et al. [15] for beef jerky dried under mid and far IR radiation. The *D*_eff_ values obtained in this study are greater than those reported in previous research on hot air drying of meat product such kaddid [57], eland jerky [58], chicken breast meat [59], and beef [60]. According to Li et al. [15], the selective heating of water molecules by the IR radiation causes the immobilized water in the myofibrillar network to migrate out of the network. The migration of the immobilized water out of the myofibrillar network increases the internal free water content, consequently expediting the transport of the free water to the surface by diffusion.

The IR power level caused significant (*p* ≤ 0.05) variations in *D*_eff_ under all drying conditions. The temperature of the drying air only caused significant variations between 30 and 40 °C, whereas the drying air velocity had a significant effect on the *D*_eff_ at IR emitter power levels of 1000 and 750 W but not at 500 W. Khir et al. [61] reported that *D*_eff_ increases with an increase in sample temperature during IR drying. The effect of the IR power, the temperature, and the velocity of the drying air on the *D*_eff_ in this study is attributed to their influence on the core temperature of the beef sample.

### 3.6. Activation Energy

**Figure 5:**
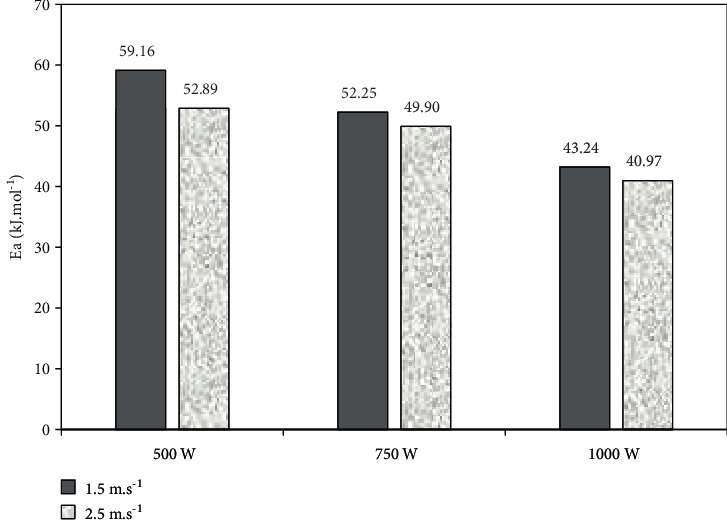
Activation energy of marinated beef under different IRHAD experimental conditions.

The activation energy (Ea) ranged between 40.97 and 59.16 kJ∙mol^−1^ (Figure 5). The Ea obtained in this study is comparable to the Ea of 32.8 kJ∙mol^−1^ obtained by Li et al. [15] during mid- and far-IR drying of beef. The difference between the Ea obtained in this study and that reported by Li et al. [15] may be due to the difference in the range of IR wavelength used in both studies. The wavelength of the IR radiation used by Li et al. [15] ranged between 2.9–3.1 and 5.8–6.2 *μ*m, whereas the wavelength of the IR radiation used in study ranged between 5.38 ± 0.17 and 12.73 ± 0.43 *μ*m. The IR radiation wavelengths used by Li et al. [15] can effectively heat up the water molecules due to their proximity to the maximum absorption wavelengths of water of 3 *μ*m and 6 *μ*m [9]. Consequently, the availability of free water in the food sample increases. Increased availability of free water in the food matrix is associated with reduced Ea [62].

The inverse relationship between the Ea and the power level of the IR emitter observed in this study is attributed to the change in the wavelength of the IR radiation. Increasing the power of the IR emitter shortens the wavelength of the IR radiation from 12.73 ± 0.43 to 5.38 ± 0.17 *μ*m. The absorption of the IR radiation by the water molecules in the beef sample increases as the radiation wavelength decreases towards the maximum absorption wavelength of water of 6 *μ*m, thus lowering the activation energy.

### 3.7. Selection of the Best Thin-Layer Drying Model

The average coefficient of determination and the average root mean square error for the five thin-layer drying models considered in this study are shown in Table 6. All the five models had an *R*^2^ > 0.9977 and a RMSE < 0.0177. The high *R*^2^ and the low RMSE indicate that all the five models can predict the changes in MR of marinated beef subjected to IRHAD with acceptable accuracy [63]. Overall, the two-term model had the highest *R*^2^ (0.9982-0.9993) and the lowest RMSE (0.0062-0.0099) across all the IRHAD conditions. Therefore, the two-term model is the most suitable model in predicting the drying behaviour of marinated beef subjected to IRHAD.

A summary of the two-term model coefficients and the statistical parameters for all the experimental drying conditions is presented in Table 7.

**Figure 6:**
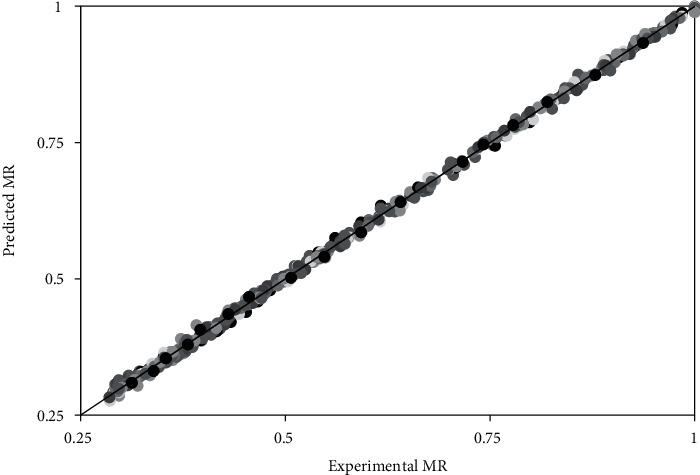
Comparison between the MR predicted by the two-term model and the experimental MR for all the drying conditions.

The plot of the experimental MR and the MR predicted using the two-term model is shown in Figure 6. The data points lie along the 45° line which confirms the high degree of agreement between the experimental and predicted MR values.

## 4. Conclusion

The IRHAD of marinated beef is significantly affected by the power level of the IR emitter, the temperature, and the velocity of the drying air. The power level of the IR emitter is the predominant factor that influences the drying process of marinated beef subjected to IRHAD. The power level of the IR emitter determines the temperature of the emitter, thus influencing the core temperature of the marinated beef sample. The drying air temperature and drying air velocity have a direct and inverse relationship, respectively, with IR emitter temperature and the core temperature of the beef samples. The core temperature of the beef sample is the most critical parameter that influences the drying kinetics of marinated beef subjected to IRHAD. The combined effect of the power level of the IR emitter, temperature, and velocity of the drying air on the drying time, drying rate, effective moisture diffusivity, and the activation energy is directly related to how these factors influence the core temperature of the beef sample. The drying of marinated beef at IR power levels of 500 W and 750 W has a short rising rate drying period followed by the falling rate drying period. The drying process at 1000 W has a short constant rate period sandwiched between the rising rate and the first falling rate period. The rising and falling rate drying periods imply that the moisture transport during IRHAD of marinated occurs partly by surface evaporation and predominantly by diffusion. The two-term thin-layer drying model best describes the drying of marinated beef under IRHAD. The effective moisture diffusivity ranges from 4.560 × 10^−10^ m^2^∙s^−1^ to 13.7 × 10^−10^ m^2^∙s^−1^, while the activation energy ranges between 40.97 and 59.16 kJ∙mol^−1^. The results from this study can inform the application of IRHAD in biltong processing. A power level of 1000 W with an IR radiation wavelength of 5.39 ± 0.17 *μ*m resulted in the shortest drying time, the highest effective moisture diffusivity, and the lowest activation energy. The power level of the IR emitter of 1000 W combined with a temperature and velocity of the drying air of 40°C and 1.5 m∙s^−1^, respectively, highly improved the drying kinetics of biltong, hence is recommended as a possible drying alternative for biltong processing.

## Figures and Tables

**Table 1:** Thin-layer drying models.

S/N	Thin-layer model	Equation	References
1	Approximation of diffusion model (ADM)	MR = *a*exp(−*kt*) + (1 − a)exp(−*kbt*)	Cherono [17]
2	Logarithmic model	MR = *a*exp(−*kt*) + *c*	Wang et al. [36]
3	Midilli model	MR = exp(−*k*(*t*^*n*^)) + *b* × *t*	Midilli et al. [37]
4	Page model	MR = exp(−*kt*^*n*^)	Ertekin and Firat [34]
5	Two-term model	MR = *a*exp(−*k*_1_*t*) + *b*exp(−*k*_2_*t*)	Erbay and Icier [38]

**Table 2:** Temperature of the IR emitter.

Drying air temperature (°C)	Drying air velocity (m∙s^−1^)	IR emitter temperature (°C)		
		1000 W	750 W	500 W
30	1.5 1.5 1.5	543.30 ± 8.73^k^	396.16 ± 5.39^g^	233.10 ± 2.61^c^
35		548.21 ± 7.36^k^	407.60 ± 5.23^h^	232.19 ± 3.1^bc^
40		566.51 ± 3.6^l^	426.42 ± 6.09^i^	237.59 ± 2.05^c^

30	2.5 2.5 2.5	523.98 ± 4.47^j^	346.65 ± 9.85^d^	220.63 ± 1.46^a^
35		524.49 ± 5.32^j^	361.69 ± 9.12^e^	219.03 ± 2.62^a^
40		527.21 ± 6.169^j^	374.10 ± 12.17^f^	222.80 ± 2.62^ab^

Means within a column followed by the same letter are not significantly different according to Fisher's unprotected least significant difference test (*p* < 0.05).

**Table 3:** Average core temperature of the beef sample.

Drying air temperature (°C)	Drying air velocity (m∙s^−1^)	Core temperature (°C)		
		1000 W	750 W	500 W
30	1.5 1.5 1.5	47.23 ± 0.26^l^	35.57 ± 0.20^e^	31.74 ± 0.07^b^
35		47.91 ± 0.22^m^	43.29 ± 0.15^j^	34.04 ± 0.05^d^
40		51.16 ± 0.36^n^	44.97 ± 0.19^k^	36.80 ± 0.18^g^

30	2.5 2.5 2.5	42.59 ± 0.26^i^	31.73 ± 0.26^b^	30.62 ± 0.08^a^
35		42.90 ± 0.30^ij^	39.38 ± 0.54^h^	32.60 ± 0.06^c^
40		47.74 ± 0.41^m^	42.83 ± 0.42^i^	36.15 ± 0.28^f^

Means within a column followed by the same letter are not significantly different according to Fisher's unprotected least significant difference test (*p* < 0.05).

**Table 4:** Summary of the drying time for marinated beef at the selected drying conditions.

Drying air temperature (°C)	Drying air velocity (m∙s^−1^)	Drying time (h)		
		1000 W	750 W	500 W
30	1.5 1.5 1.5	7.17 ± 0.17^c^	11.72 ± 0.25^f^	15.00 ± 0.17^i^
35		7.11 ± 0.10^c^	8.11 ± 0.19^d^	13.00 ± 0.17^g^
40		5.61 ± 0.35^a^	8.10 ± 0.24^d^	11.83 ± 0.165^f^

30	2.5 2.5 2.5	8.11 ± 0.19^d^	14.50 ± 0.50^i^	16.22 ± 0.25^j^
35		8.22 ± 0.19^d^	10.06 ± 0.10^e^	13.94 ± 0.42^h^
40		6.50 ± 0.17^b^	8.39 ± 0.19^d^	11.78 ± 0.25^f^

Means within a column followed by the same letter are not significantly different according to Fisher's unprotected least significant difference test (*p* < 0.05).

**Table 5:** Effective moisture diffusivity of marinated beef during IRHAD.

Drying air temperature (°C)	Drying air velocity (m∙s^−1^)	*D*_eff_ (m^2^∙s^−1^)		
		1000 W	750 W	500 W
30	1.5 1.5 1.5	11.4E-10^d^	6.85E-10^b^	4.56E-10^a^
35		11.4E-10^d^	9.13E-10^c^	6.85E-10^b^
40		13.7E-10^e^	9.13E-10^c^	6.85E-10^b^

30	2.5 2.5 2.5	9.13E-10^c^	4.56E-10^a^	4.56E-10^a^
35		9.13E-10^c^	6.85E-10^b^	6.85E-10^b^
40		11.4E-10^d^	9.13E-10^c^	6.85E-10^b^

Means within a column followed by the same letter are not significantly different according to Fisher's unprotected least significant difference test (*p* < 0.05).

**Table 6:** The average *R*^2^ and RMSE for the thin-layer models at different IR power levels.

S/No	Model	*R* ^2^			RMSE		
		500 W	750 W	1000 W	500 W	750 W	1000 W
1	Two-term	0.9993	0.9982	0.9993	0.0062	0.0099	0.0065
2	Approximation of diffusion	0.9989	0.9981	0.9992	0.0080	0.0099	0.0057
3	Midilli	0.9978	0.9980	0.9988	0.0177	0.0104	0.0085
4	Page	0.9977	0.9960	0.9975	0.0109	0.0143	0.0118
5	Logarithmic	0.9977	0.9964	0.9961	0.0107	0.0139	0.0141

**Table 7:** Model coefficient and statistical parameters for the two-term thin-layer drying model.

IR power	T (°C)	Air vel (m∙s^−1^)	Model coefficient				*R* ^2^	RMSE
			*K*	K2	*a*	*b*		
500 W	30	1.5	0.7236	0.0686	0.1444	0.8566	0.9992	0.0063
	30	2.5	0.2642	0.0459	0.3569	0.6446	0.9987	0.0084
	35	1.5	0.3293	0.0755	0.2384	0.7719	0.9988	0.0086
	35	2.5	0.1039	-0.155	0.9913	0.0070	0.9997	0.0042
	40	1.5	0.4083	0.0671	0.3456	0.6629	0.9998	0.0038
	40	2.5	0.7276	0.0808	0.2245	0.7813	0.9994	0.0057
	Mean						0.9993	0.0062

750 W	30	1.5	0.0560	0.4278	0.6141	0.3766	0.9991	0.0069
	30	2.5	0.5176	0.0532	0.3654	0.6472	0.9992	0.0067
	35	1.5	1.094	0.124	0.2192	0.77	0.9978	0.0116
	35	2.5	0.0568	0.3687	0.5287	0.4783	0.9992	0.0071
	40	1.5	0.2071	-0.2568	0.9406	0.0135	0.9948	0.0188
	40	2.5	0.7417	0.1078	0.3	0.6899	0.9990	0.0080
	Mean						0.9982	0.0099

1000 W	30	1.5	0.0368	0.4232	0.3359	0.6652	0.9996	0.0053
	30	2.5	0.114	1.129	0.7512	0.2458	0.9994	0.0061
	35	1.5	1.428	0.1494	0.2012	0.8051	0.9994	0.0063
	35	2.5	0.4459	0.0746	0.4666	0.5225	0.9995	0.0057
	40	1.5	0.1733	1.32	0.774	0.235	0.9987	0.0093
	40	2.5	1.434	0.1364	0.2524	0.7504	0.9993	0.0064
	Mean						0.9993	0.0065

## Data Availability

The data is available on request through the corresponding author.

## References

[1] Muga F., Workneh T., Marenya M. (2020). Modelling the thin-layer drying of beef biltong processed using hot air drying. *Journal of Biosystems Engineering*.

[2] Dzimba F. E. J., Faria J., Walter E. H. M. (2007). Testing the sensory acceptability of biltong formulated with different spices. *African Journal of Agricultural Research*.

[3] Nortjé K., Buys E., Minnaar A. (2005). Effect of *γ*-irradiation on the sensory quality of moist beef biltong. *Meat Science*.

[4] Naidoo K., Lindsay D. (2010). Survival of Listeria monocytogenes, and enterotoxin-producing Staphylococcus aureus and Staphylococcus pasteuri, during two types of biltong-manufacturing processes. *Food Control*.

[5] Saayman M. (2015). *Biltong of Great Value to South African Economy*.

[6] Attwell E. (2003). Biltong wakes up. *Food Reviews*.

[7] Henning M., Hagedorn-Hansen D., Von Leipzig K. H. (2018). A conceptual framework to increase competitiveness in a biltong factory. *South African Journal of Industrial Engineering*.

[8] Xie X., Li X., Zhang C. (2013). Combined mid-infrared and hot air drying reduces energy-consumption and improves quality of jerky. *Transactions of the Chinese Society of Agricultural Engineering*.

[9] Aboud S. A., Altemimi A. B., Al-HiIphy R. S., Yi-Chen A. L., Cacciola F. (2019). A comprehensive review on infrared heating applications in food processing. *Molecules*.

[10] Soydan Karabacak M., Esin A., Cekmecelioglu D. (2014). Drying behavior of meat samples at various fiber directions and air conditions. *Drying Technology*.

[11] Bellagha S., Sahli A., Farhat A., Kechaou N., Glenza A. (2007). Studies on salting and drying of sardine (Sardinella aurita): experimental kinetics and modeling. *Journal of Food Engineering*.

[12] Duan Z.-h., Jiang L.-n., Wang J.-l., Yu X.-y., Wang T. (2011). Drying and quality characteristics of tilapia fish fillets dried with hot air-microwave heating. *Food and Bioproducts Processing*.

[13] Kowalski S. J., Mierzwa D. (2009). Convective drying in combination with microwave and IR drying for biological materials. *Drying Technology*.

[14] Rastogi N. K. (2012). Recent trends and developments in infrared heating in food processing. *Critical Reviews in Food Science and Nutrition*.

[15] Li X., Xie X., Zhang C.-h., Zhen S., Jia W. (2018). Role of mid- and far-infrared for improving dehydration efficiency in beef jerky drying. *Drying Technology*.

[16] Cherono K., Mwithiga G., Schmidt S. (2016). Infrared drying as a potential alternative to convective drying for biltong production. *Italian journal of food safety*.

[17] Cherono K. (2014). *Infrared Drying of Biltong*.

[18] Afzal T., Abe T., Hikida Y. (1999). Energy and quality aspects during combined FIR-convection drying of barley. *Journal of Food Engineering*.

[19] Hebbar H. U., Vishwanathan K., Ramesh M. (2004). Development of combined infrared and hot air dryer for vegetables. *Journal of Food Engineering*.

[20] Trujillo F. J., Wiangkaew C., Pham Q. T. (2007). Drying modeling and water diffusivity in beef meat. *Journal of Food Engineering*.

[21] Abe T., Afzal T. (1997). Thin-layer infrared radiation drying of rough rice. *Journal of Agricultural Engineering Research*.

[22] Das I., Das S., Bal S. (2004). Drying performance of a batch type vibration aided infrared dryer. *Journal of Food Engineering*.

[23] Toğrul H. (2005). Simple modeling of infrared drying of fresh apple slices. *Journal of Food Engineering*.

[24] Puente-Díaz L., Ah-Hen K., Vega-Gálvez A., Lemus-Mondaca R., Scala K. D. (2013). Combined infrared-convective drying of murta (Ugni molinae Turcz) berries: kinetic modeling and quality assessment. *Drying Technology*.

[25] Sui Y., Yang J., Ye Q., Li H., Wang H. (2014). Infrared, convective, and sequential infrared and convective drying of wine grape pomace. *Drying Technology*.

[26] Sadin R., Chegini G., Khodadadi M. (2017). Drying characteristics and modeling of tomato thin layer drying in combined infrared-hot air dryer. *Agricultural Engineering International: CIGR Journal*.

[27] Doymaz İ. (2012). Infrared drying of sweet potato (Ipomoea batatas L.) slices. *Journal of Food Science and Technology*.

[28] Feyissa A. H., Adler-Nissen J., Gernaey K. Model of heat and mass transfer with moving boundary during roasting of meat in convection-oven.

[29] AOAC (2012). *Official Methods of Analysis of AOAC International*.

[30] Jones M. (2017). *Profiling of Traditional South African Biltong in Terms of Processing, Physicochemical Properties and Microbial Stability during Storage*.

[31] Strydom P., Zondagh B., Dikeman M., Devine C. (2014). Biltong: a major South African ethnic meat product. *Encyclopaedia of meat sciences*.

[32] von Gersdorff G. J., Porley V. E., Retz S. K., Hensel O., Crichton S., Sturm B. (2018). Drying behavior and quality parameters of dried beef (biltong) subjected to different pre-treatments and maturation stages. *Drying Technology*.

[33] Elstein (2014). *Ceramic Infrared Emitters*.

[34] Ertekin C., Firat M. Z. (2015). A comprehensive review of thin-layer drying models used in agricultural products. *Critical Reviews in Food Science and Nutrition*.

[35] Zhu A., Shen X. (2014). The model and mass transfer characteristics of convection drying of peach slices. *International Journal of Heat and Mass Transfer*.

[36] Wang Z., Sun J., Liao X. (2007). Mathematical modeling on hot air drying of thin layer apple pomace. *Food Research International*.

[37] Midilli A., Kucuk H., Yapar Z. (2007). A new model for single-layer drying. *Drying Technology*.

[38] Erbay Z., Icier F. (2010). A review of thin layer drying of foods: theory, modeling, and experimental results. *Critical Reviews in Food Science and Nutrition*.

[39] Ali S. Z., De Luca A., Hopper R., Boual S., Gardner J., Udrea F. (2015). A low-power, low-cost infra-red emitter in CMOS technology. *IEEE Sensors Journal*.

[40] Ott T., Schossig M., Norkus V., Gerlach G. (2015). Efficient thermal infrared emitter with high radiant power. *Journal of Sensors and Sensor Systems*.

[41] Das I., Das S. K., Pan Z., Atungulu G. G. (2010). Emitters and Infrared heating system design. *Infrared Heating for Food and Agricultural Processing*.

[42] Ratti C., Mujumdar A. S., Mujumdar A. S. (2007). Infrared drying. *Handbook of Industrial Drying*.

[43] Pan Z., Atungulu G., Li X., Sun D.-W. (2014). Infrared heating. *Emerging Technologies for Food Processing*.

[44] Riadh M. H., Ahmad S. A. B., Marhaban M. H., Soh A. C. (2015). Infrared heating in food drying: an overview. *Drying Technology*.

[45] Onwude D. I., Hashim N., Abdan K., Janius R., Chen G. (2019). The effectiveness of combined infrared and hot-air drying strategies for sweet potato. *Journal of Food Engineering*.

[46] Kocabiyik H., Tezer D. (2009). Drying of carrot slices using infrared radiation. *International Journal of Food Science & Technology*.

[47] Jones M., Arnaud E., Gouws P., Hoffman L. C. (2017). Processing of South African biltong–a review. *South African Journal of Animal Science*.

[48] Sharma G., Verma R., Pathare P. (2005). Thin-layer infrared radiation drying of onion slices. *Journal of Food Engineering*.

[49] Kumar D. P., Hebbar H. U., Ramesh M. (2006). Suitability of thin layer models for infrared-hot air-drying of onion slices. *LWT-Food Science and Technology*.

[50] Nasiroglu S., Kocabiyik H. (2009). Thin-layer infrared radiation drying of red pepper slices. *Journal of Food Process Engineering*.

[51] Srikiatden J., Roberts J. S. (2007). Moisture transfer in solid food materials: a review of mechanisms, models, and measurements. *International Journal of Food Properties*.

[52] Jaturonglumlert S., Kiatsiriroat T. (2010). Heat and mass transfer in combined convective and far-infrared drying of fruit leather. *Journal of Food Engineering*.

[53] Kocabiyik H., Pan Z., Atungulu G. G. (2010). Combined infrared and hot air drying. *Infrared heating for food and agricultural processing*.

[54] Mongpraneet S., Abe T., Tsurusaki T. (2002). Accelerated drying of welsh onion by far infrared radiation under vacuum conditions. *Journal of Food Engineering*.

[55] Karel M., Lund D. B. (2003). *Physical Principles of Food Preservation*.

[56] Feyissa A. H., Gernaey K. V., Adler-Nissen J. (2013). 3D modelling of coupled mass and heat transfer of a convection-oven roasting process. *Meat Science*.

[57] Chabbouh M., Sahli A., Bellagha S. (2013). Does the spicing step affect the quality and drying behaviour of traditional kaddid, a Tunisian cured meat?. *Journal of the Science of Food and Agriculture*.

[58] Kucerova I., Hubackova A., Rohlik B.-A., Banout J. (2015). Mathematical modeling of thin-layer solar drying of Eland (Taurotragus oryx) Jerky. *International Journal of Food Engineering*.

[59] Ismail O. (2017). An experimental and modeling investigation on drying of chicken meat in convective dryer. *Studia Universitatis Babes-Bolyai Chemia*.

[60] Mewa E. A., Okoth M. W., Kunyanga C. N., Rugiri M. N. (2018). Drying modelling, moisture diffusivity and sensory quality of thin layer dried beef. *Current Research in Nutrition and Food Science Journal*.

[61] Khir R., Pan Z., Salim A., Hartsough B. R., Mohamed S. (2011). Moisture diffusivity of rough rice under infrared radiation drying. *LWT-Food Science and Technology*.

[62] Hwang S.-S., Cheng Y.-C., Chang C., Lur H.-S., Lin T.-T. (2009). Magnetic resonance imaging and analyses of tempering processes in rice kernels. *Journal of Cereal Science*.

[63] Nguyen T. V. L., Nguyen M. D., Nguyen D. C., Bach L. G., Lam T. D. (2019). Model for thin layer drying of lemongrass (Cymbopogon citratus) by hot air. *Processes*.

